# Polarized Light Field Imaging for Single-Shot Reflectance Separation †

**DOI:** 10.3390/s18113803

**Published:** 2018-11-06

**Authors:** Jaewon Kim, Abhijeet Ghosh

**Affiliations:** Imperial College London, London SW7 2AZ, UK; jaewon.kim15@imperial.ac.uk

**Keywords:** light-field, polarization, reflectance separation, single-shot

## Abstract

We present a novel computational photography technique for single-shot separation of diffuse/specular reflectance, as well as novel angular domain separation of layered reflectance. We present two imaging solutions for this purpose: two-way polarized light-field (TPLF) imaging and four-way polarized light-field (FPLF) imaging. TPLF imaging consists of a polarized light-field camera, which simultaneously captures two orthogonal states of polarization. A single photograph of a subject acquired with the TPLF camera under polarized illumination then enables standard separation of diffuse (depolarizing) and polarization preserving specular reflectance using light-field sampling. We further demonstrate that the acquired data also enable novel angular separation of layered reflectance including separation of specular reflectance and single scattering in the polarization preserving component, as well as separation of shallow scattering from deep scattering in the depolarizing component. FPLF imaging further generalized the functionality of TPLF imaging under uncontrolled unpolarized or partially polarized illumination such as outdoors. We apply our approach for efficient acquisition of facial reflectance including diffuse and specular normal maps and novel separation of photometric normals into layered reflectance normals for layered facial renderings. We validate our proposed single-shot layered reflectance separation under various imaging conditions and demonstrate it to be comparable to an existing multi-shot technique that relies on structured lighting while achieving separation results under a variety of illumination conditions.

## 1. Introduction

Accurate modelling and realistic reproduction of appearance has been widely explored in computer vision and graphics. There have been significant advances in digital photography over the last decades, which have made measurement-based appearance modelling popular for various applications in realistic computer graphics. Especially, the method is well aligned with realistic appearance modelling of human faces, enabling considerable applications in the entertainment (such as movies, games and advertisement), medical (dermatology) and beauty (cosmetics) sectors. Over the years, researchers have presented various measurement-based modelling techniques and setups for acquiring facial and material reflectance [1,2,3]. Practically, however, the measured reflectance data need to be fit to suitable analytic or physics-based reflectance models in a rendering pipeline. This practice of fitting between measurements and appropriate models has driven the separation of individual reflectance components, such as specular (surface) and diffuse (subsurface), which has become important in measurement-based modelling approaches. Researchers have thus proposed various reflectance separation techniques, particularly for dielectric materials, based on polarized and spectral information [4,5,6]. Among these, polarization-based separation in conjunction with a sophisticated light stage setup [7] has achieved high quality separation of specular and diffuse reflectance and has been applied as one of the optimal solutions for high-end capture of human faces and objects. However, this approach has traditionally required multi-shot capture with different polarization modes (such as cross- and parallel polarization states) for separate acquisition of reflectance components or optically complicated imaging systems (e.g., multiple cameras sharing an optical axis through a beam-splitter) for multiplexed acquisition of different polarization states. This polarization-based approach has also been extended for a more challenging task, fine-grained separation of layered reflectance (e.g., in skin), combining the computational illumination technique with a sophisticated multi-shot acquisition [8].

In this paper, we present an extension of our recent work [9] proposing two novel techniques for efficient single-shot acquisition of surface and subsurface reflectance with novel computational analysis of the polarized light-field data for layered reflectance separation. The work in [9] proposed two-way polarized light-field (TPLF) imaging, which was implemented by a low-resolution light-field camera, attaching two polarization filters. In this paper, we present an extended version of the polarized light-field technique, four-way polarized light-field (FPLF) imaging, with higher polarization dimensions and imaging resolution for more general applications of single-shot reflectance acquisition. Our two techniques achieve not only standard diffuse-specular separation, but also separation of layered reflectance albedos spanning specular, single scattering, shallow scattering and deep scattering by a single-shot light-field image (see Figure 7). While the TPLF technique required uniform polarized illumination at a fixed angle, the FPLF technique achieves high-quality reflectance separation under unpolarized or partially polarized illumination at an arbitrary angle. Besides albedo estimation, the two techniques can be applied for acquiring photometric diffuse and specular normal maps and further photometric normal maps of layered reflectance with multiple shots under polarized spherical gradient illumination for realistic skin rendering applications.

To summarize, the specific technical contributions of this work are as follows:

- The practical realization of TPLF imaging by attaching orthogonal linear polarizers and FPLF imaging by attaching four linear polarizers at 0, 45, 90 and 135${}^{\circ }$, respectively, to the aperture of the main lens of a commercial light-field camera. TPLF imaging angularly multiplexes two orthogonal polarization states of incoming rays in a single-shot and enables separation of diffuse and specular reflectance using angular light-field sampling under controlled polarized illumination. FPLF imaging enables a similar function under uncontrolled unpolarized or partially polarized illumination such as outdoor illumination.
- A novel computational method for single-shot separation of layered reflectance by analysing the acquired light-field data in the 2D angular domain. The method allows us to estimate layered reflectance albedos using a single-shot polarized light-field image under either uniform (TPLF) or partially (FPLF) polarized illumination. Furthermore, the method achieves novel layered separation of photometric normals acquired under polarized spherical gradient illumination.

The rest of the paper is organized as follows: Section 2 describes reviews of related work in three categories before presenting our proposed TPLF camera and computational method of diffuse-specular separation in Section 3. Section 4 presents the extension to four-way polarized light-field imaging. Section 5 then describes our novel computational approach to acquire separated layered albedos and photometric normals of facial reflectance by analysing polarized light-field imagery. We conclude with detailed analysis and limitations of our technique in Section 8 after presenting validation experiments and facial rendering applications using photometric normals of layered reflectance in Section 7.

## 2. Related Work

We classify relevant previous work into three categories: reflectance separation, light-field imaging and facial reflectance capture. A brief review of each category is as follows.

### 2.1. Reflectance Separation

In computer vision and graphics, acquisition of separated reflectance components (such as specular and diffuse) has long been of interest for various applications such as photometric stereo, reflectometry and realistic facial rendering. The colour property of dielectric materials depends on the characteristics of diffuse (subsurface) reflectance rather than specular (surface) reflectance. Hence, colour-based approaches [10] could be applied for separating diffuse and specular reflectance. However, those approaches have limited performance for textured objects, which could be addressed by combining polarization imaging [4]. Alternatively, a colour space transform could be utilized for photometric stereo applications by selectively eliminating the specular reflectance component [5]. This approach allows preserving the diffuse component by removing the specular component without explicitly estimating the diffuse colour for dichromatic materials. The polarization imaging method, proposed by Debevec et al. [6], successfully separates diffuse and specular reflectance of a human face. The method acquires cross- and parallel polarization states illuminating a face with a polarized point light and fits the separated reflectance images to a microfacet BRDF model. Nayar et al. [11] proposed a computational illumination technique to separate direct and indirect (global) reflectance of a general scene, which acquires a series of photographs while sequentially emitting high frequency patterns in a phase shifted manner. A related approach [12] also separates reflectance of a general scene by utilizing high frequency environmental illumination in an angularly-shifted phase. More recently, Tunwattanapong et al. [13] extended the computational illumination technique with (higher order) spherical harmonic illumination for acquiring both separation and estimation of reflectance. As another extension, specific patterns designed with Fourier basis functions were utilized for separating specular and diffuse component in the frequency domain [14]. However, those methods have limited applications to only stationary scenes since they require multiple shots to separate reflectance using computational illumination. Instead, we propose a single-shot acquisition method for reflectance separation under a fixed illumination condition, which can also be applied to dynamic scenes. Ghosh et al. [15] proposed circularly-polarized reflectometry for separating diffuse and specular reflectance, which calculates the complete Stokes parameters of reflected polarization using circularly-polarized spherical illumination. Specular highlights can be removed by a similar approach [16], which estimates the polarization angle of the specular highlight by measuring linear Stokes parameters. Recently, Riviere et al. [17] have employed similar linear polarization measurements under uncontrolled outdoor illumination for estimating surface reflectance of planar surfaces. However, these approaches require multiple shots with rotating polarizers at a specific angle in front of an imaging device to measure the Stokes parameters. In contrast, our proposed approach exploits an off-the-shelf light-field camera with fixed linear polarizers attached in its entrance pupil for separating various reflectance components based on single-shot polarization imaging.

### 2.2. Light Field and Multimodal Imaging

Acquisition of the 4D light-field, typically defined in the 2D spatial + 2D angular domain, has been extensively researched with various ways such as a lenslet array [18] or a patterned mask film [19]. Many applications of light-field have been developed including synthetic refocusing [18], glare reduction [20] and descattering through a participating media [21]. Narasimhan et al. [22] proposed a multidimensional imaging method using a custom-designed Bayer pattern for enhanced imagery of a high dynamic range or multi-spectrum. They also tried to augment image resolution by estimating subpixels through a learning algorithm. In relation to our method, some have proposed multimodal imaging through a pinhole-based [23] or mirror-based [24] light-field camera attached with specific optical filters at the aperture plane. While these approaches can acquire multimodal light information such as multiple spectra and polarization, they are less practical due to requiring sophisticated implementation on an optical table. Recently, Tao et al. [25] proposed a specularity removal approach using the point-consistency assumption over angular samples of a scene point. The point-consistency assumption holds in the diffuse region, but not the specular region, and hence, it can be exploited for separating the two regions. However, the approach has limited performance for the saturated pixels (bright central parts) of a specular region where the assumption of point-consistency also holds as in the diffuse region. Compared to the above approaches, we propose an optimized light-field imaging for single-shot acquisition of multiple polarization states with a practical implementation consisting of a simple modification of a commercial light-field camera. We additionally propose a novel approach of layered reflectance separation, which has not been previously addressed with light-field imaging.

### 2.3. Facial Reflectance Capture

Debevec et al. [6] presented a light stage to effectively acquire a four-dimensional reflectance field over a human face for relighting tasks. The method achieves photorealistic facial renderings using a view-dependent reflectance field generated by processing large data consisting of 150–200 photographs. Weyrich et al. [26] produced a dense reflectance field of a face with a similar light stage by acquiring multiple (16) views and densely sampling the incident illumination at 150 directions. Their method combines multiview analysis with intensity-based reflectance separation over a facial surface. The acquired data are fit to appropriate models of specular and diffuse reflectance for various facial rendering applications. While the method can produce high quality facial rendering from arbitrary viewpoints, its practicality is limited due to requiring large amounts of data.

A recent photometric stereo method, presented by Ma et al. [27], achieves high-quality facial reflectance and surface normal maps using four spherical illumination patterns with a light stage. Their method further produces separated albedos and surface normal maps of specular and diffuse reflectance by using spherical illumination in cross- and parallel polarization states. The separated specular and diffuse normal maps are combined with a hybrid normal mapping technique, which allows realistic facial renderings. Ghosh et al. [8] extended Ma’s acquisition system and proposed a practical acquisition method of layered facial reflectance using a limited number of measurements. They modelled the facial layered reflectance as four components: specular reflectance, single scattering, shallow scattering and deep scattering. The specular reflectance and single scattering were acquired by a fitting process in the polarization preserving component combining polarized illumination of the spherical gradient and frontal point source. Then, shallow and deep scattering were further separated using the method of direct and global component separation [11] in a cross-polarization state (to exclude the polarization preserving components). While both methods, proposed by Ma and Ghosh et al., require fewer measurements than conventional facial capture techniques with a light stage, they are still multi-shot techniques requiring several seconds’ acquisition time with a DSLR camera. Ma et al.’s method acquires eight photographs under spherical gradient illumination in parallel and cross-polarization states for producing separated specular and diffuse normal maps. Ghosh et al.’s method requires six additional photographs under frontal projector illumination for obtaining albedos of layered facial reflectance. Since their method acquires reflectance albedos under different imaging conditions, an additional fitting process is required to match the albedos for a combined rendering result. Instead, our method achieves layered reflectance albedos using uniform (TPLF) or partially (FPLF) polarized illumination with just a single light-field photograph. Furthermore, our method can achieve novel photometric normals of layered reflectance with just three additional photographs.

Ma et al.’s method [27] is limited to facial capture at a single viewpoint because its reflectance separation using polarization difference imaging works for a calibrated view with polarized spherical gradient illumination. Similarly, Fyffe et al. [28] applied unpolarized spherical gradient illumination for facial performance capture at multiple views. They proposed a heuristic rule to separate facial reflectance for the rendering process. Ghosh et al.’s subsequent work [29] achieved multiview face capture with polarized spherical gradient illumination by using a pair of orthogonal linear polarization patterns (latitude-longitude) on the LED sphere that are symmetric about the vertical axis. This illumination method allows acquiring cross- and parallel polarization states at multiple viewpoints with a fixed vertical linear polarizer in front of the camera. However, it still requires multi-shot photographs to acquire two polarization modes while switching LEDs on a sophisticated illumination system. Alternatively, passive approaches with uniform illumination [30,31] could achieve facial rendering results by reconstructing facial geometry and acquiring diffuse albedo under flat illumination without explicitly separating specular and diffuse reflectance.

More recently, Fyffe and Debevec [32] have proposed a single-shot approach for separating specular and diffuse albedos and generating a surface normal map with additional spectrum dimension to conventional polarized illumination. They built a coaxial imagery system consisting of multiple cameras and a polarizing beam splitter to acquire two orthogonal polarization modes simultaneously using the latitude-longitude polarization patterns [29]. In addition, they simultaneously acquired X, Y and Z spherical gradient patterns using colour space encoding by allocating RGB LEDs to each illumination pattern. Despite the single-shot acquisition of separated reflectance and surface normals (modulo slightly lower quality), their method is less practical due to the sophisticated acquisition system requiring RGB spherical gradient illumination and a precisely aligned camera pair. An RGB LED sphere has recently also been employed by Kampouris et al. [33] for a two-shot diffuse-specular separation using binary RGB gradient illumination with useful, but sub-optimal results. They demonstrate higher quality separation under white illumination with binary gradients. However, this requires six measurements of the X, Y and Z binary gradients and complements. Instead, our approach converts a commercial light-field camera into a polarization encoding device, which allows acquiring multiple polarization modes in a single-shot for high-quality separation of reflectance albedos under various illumination conditions.

## 3. Two-Way Polarized Light-Field Imaging

**Figure 1:**
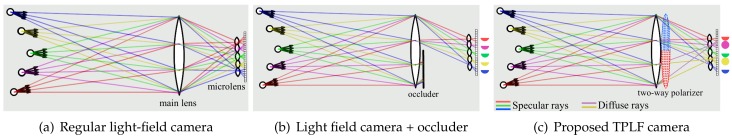
Ray simulation (based on Andrew Adams’s optical bench toolkit (http://graphics.stanford. edu/~abadams/lenstoy.swf)) for illustrating the concept of the proposed TPLF camera. (**a**) Regular light-field imaging with a camera with a microlens array for diffuse and specular rays. If a half occluder is placed behind the main lens as in (**b**), only half the rays reach the sensor. Our TPLF camera in (**c**) has the occluder replaced with a two-way polarizer, which is half vertically and half horizontally polarized ((**c**) Eurographics Association 2016 [9]).

We now describe our acquisition technique for diffuse and specular reflectance based on our novel two-way polarized light-field (TPLF) camera, which addresses the challenge of capturing two different polarization states in a single-shot. Figure 1a shows a ray diagram for a regular light-field camera. For simplicity, let us assume there are five scene points emitting four rays in diffuse or specular reflection. The four rays emitted from each point in various directions are focused into a point by the main lens and dispersed into sensor pixels by the microlens array. Let us think about a half occluder placed at the back of the main lens as shown in Figure 1b. Only half of the rays reach the camera sensor, and the other half are blocked by the occluder, resulting in the semicircular rays as shown on the right. In the case of our TPLF camera shown in Figure 1c, the occluder is replaced with a two-way linear polarizer, where the upper half is vertically polarized and the bottom half is horizontally polarized. This way, the blocked and unblocked rays in the occluder case now pass through the horizontal and the vertical polarizers, respectively. If the scene is illuminated with vertically-polarized light, specular reflection rays (illustrated in red, green and blue) can pass through only the vertically-polarized region. This results in the semicircular images via the microlens array on the right side. On the other hand, diffuse reflection rays (illustrated in purple and yellow) can pass through the entire polarizer (due to depolarization) and result in the fully-circular images on the right. Consequently, given linearly-polarized illumination, our TPLF camera provides a semicircular and a fully-circular microlens image for specular and diffuse surface reflections, respectively. These respective microlens imaging regions are predetermined once using a calibration photograph, and TPLF photographs can thereafter be automatically processed using this calibration information. Note that pixels in a raw photograph are sensed through a mosaic colour filter. Hence, we have to additionally demosaic colour pixels by averaging colour information in 4 × 4 pixel regions to generate a colour TPLF photograph.

**Figure 2:**
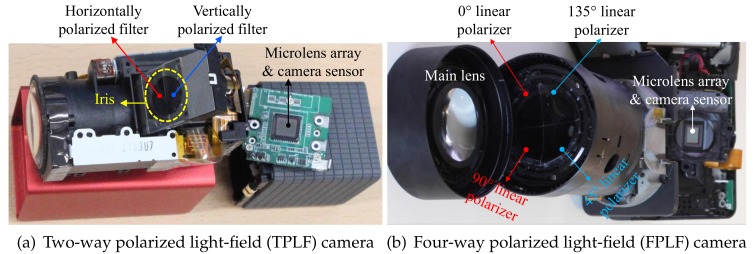
Our proposed polarized light-field cameras. (**a**) Components of the TPLF camera in disassembly employing a Lytro Red Hot camera ((**c**) Eurographics Association 2016 [9]). (**b**) Lytro Illum camera employed for FPLF imaging with four linear polarizers at 0, 45, 90 and 135°.

Figure 2a shows the actual TPLF camera used for our experiments, which was built from a Lytro Red Hot 16 GB camera to which we attached a two-way linear polarizer in the pupil plane of the main lens. The camera produces a light-field image at 1080 × 1080 resolution with a 331 × 381 microlens array. Although Lytro software and some other methods allow increasing the resolution of light-field images to higher than the number of microlenses, we only carry out single-pixel generation per each microlens image for simplicity, which means our processed images have 331 × 381 resolution.

### Diffuse-Specular Separation

**Figure 3:**
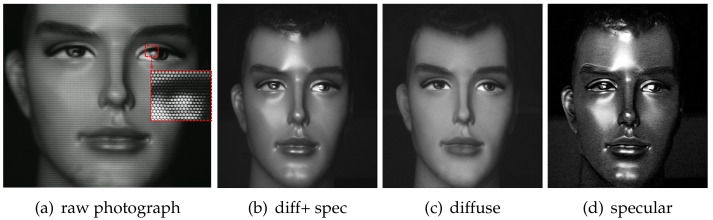
(**a**) A raw photograph of a mannequin taken with our TPLF camera. (**b**) Parallel polarized (diffuse + specular) component and separated (**c**) diffuse and (**d**) specular component images generated from the raw image. The mannequin is made of glossy plastic ((**c**) Eurographics Association 2016 [9]).

Figure 3a shows an example photograph captured by the TPLF camera of a plastic mannequin. In the inset image of the eye, each circular pattern corresponds to the contribution of rays passing through each microlens. It can be seen that the lower semi-circular region has very bright intensity, which is contributed by strong polarization preserving specular rays imaged through a microlens. As indicated in our ray simulation (Figure 1c), the upper semicircular region has a lower intensity since the specular rays are filtered out by cross-polarization in this region. Conversely, diffuse rays, which are depolarized, contribute equally to the whole circular microlens image. By exploiting this spatial separation between diffuse and specular rays, parallel polarized (diffuse + specular) and cross-polarized diffuse-only images could be generated from sampling pixels over each respective semicircular region. Here, the diffuse + specular component image (Figure 3b) was generated by averaging pixels sampled from lower semicircular regions. Likewise, a diffuse-only component image (Figure 3c) was generated by averaging pixels from the upper semicircular region. Subtraction of the diffuse-only image from the diffuse + specular image generated the specular (polarization preserving) component image, as shown in Figure 3d. Note that due to cross-polarization, the diffuse-only component (c) is imaged with only half intensity.

**Figure 4:**
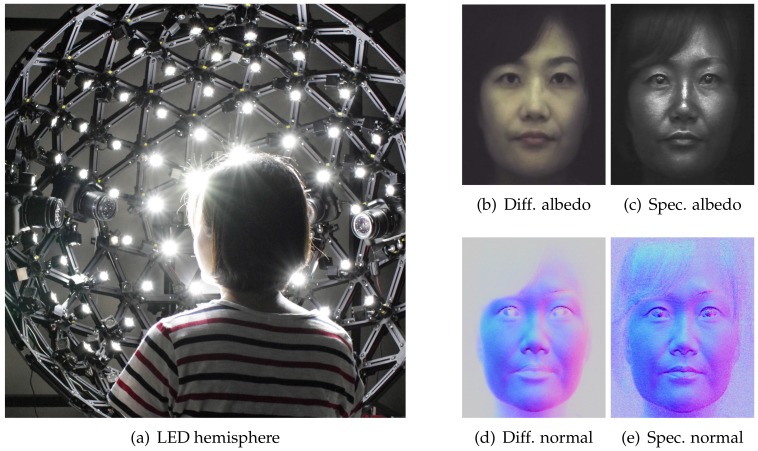
Our hemispherical illumination system and albedos and normals for separated diffuse and specular reflectance. (**a**) LED hemisphere for illuminating a subject with polarized spherical illumination. Separated diffuse (**b**,**d**) and specular (**c**,**e**) albedo and photometric normals of a female subject acquired using four measurements under polarized spherical gradient illumination ((**c**) Eurographics Association 2016 [9]).

The diffuse-specular separation examples shown in Figure 3, as well the separation result of the female subject shown in Figure 4 were acquired under uniform illumination using an LED hemisphere shown in Figure 4a. The hemispherical illumination system consists of a total of 130 LED (5 W) light sources. A linear polarizer is mounted in front of each light source and adjustable to rotate along the tangent direction to the light source. We pre-calibrate the polarizer orientation on all the lights to cross-polarize with respect to a frontal camera viewing direction similar to the procedure described in [27]. Besides recording the reflectance of a subject under constant uniform illumination to estimate the albedo, the LED hemisphere also allows us to record the subject’s reflectance under polarized spherical gradient illumination in order to estimate photometric normals. Figure 4b–e shows separated diffuse and specular normals estimated for the female subject shown in Figure 4 using three additional measurements under the X, Y and Z spherical gradients. As can be seen, the diffuse normal is smooth due to blurring of surface detail due to subsurface scattering, while the specular normal contains high frequency skin mesostructure detail due to first surface reflection [27]. However, we only need to make a total of four measurements for separated albedo and photometric normals, as we do not need to flip a polarizer in front of the camera [27] or switch polarization on the LED sphere [29] in order to observe two orthogonal polarization states.

## 4. Four-Way Polarized Light-Field Imaging

**Figure 5:**
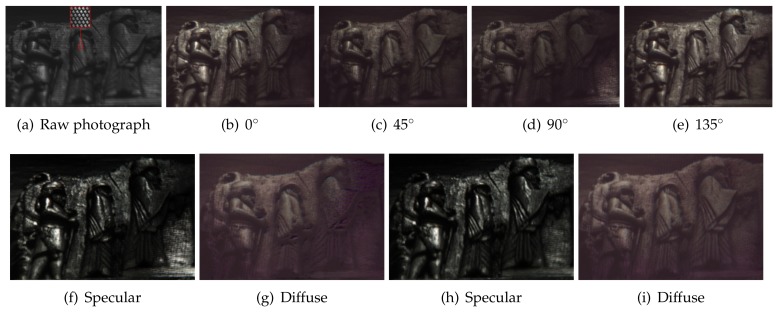
Single-shot separation of diffuse and specular reflectance under uncontrolled outdoor illumination (sunlight) using the proposed four-way polarized light-field (FPLF) camera. (**a**) A raw photograph of a piece of Persian relief taken with our FPLF camera. (**b**–**e**) Images sensed by rays travelling through each polarizer, which are generated by a single-shot light-field photograph. (**f**,**g**) Specular and diffuse albedo acquired by processing the three polarization images in (**b**–**d**). (**h**,**i**) Specular and diffuse albedo acquired by processing the four polarization images in (**b**–**e**).

This section describes our novel four-way polarized light-field (FPLF) camera, which extends a TPLF camera with four linear polarizers. Figure 2b shows our FPLF camera, which was built from a Lytro Illum camera by attaching four linear polarizers at 0, 45, 90 and 135${}^{\circ }$ in the pupil plane of the main lens. The camera can produce a light-field image at a maximum 2450 × 1634 resolution with a 539 × 432 microlens array using Lytro’s official software, Lytro Desktop. The raw sensor resolution of the camera is 7728 × 5368 pixels. Our sampling algorithm produces a light-field image at 1078 × 864 resolution, which results in two pixel sampling per each microlens region. We found this sampling to yield a high SNR with suitable spatial details. Section 3 describes how two images under vertical and horizontal polarization states are obtained by separately sampling pixels in the upper and lower semicircular region of a TPLF photograph. Similarly, we can obtain four images under different polarization states at 0, 45, 90 and 135${}^{\circ }$ by separately sampling each quarter region of a FPLF photograph. Figure 5a shows a raw FPLF photograph over a piece of Persian relief made of polished stone acquired under sunlight. The inset image of (a) clearly shows two quarter-circular regions in the upper part of each microlens image. Since the polarization angle of incident light is closely aligned with the polarization filter angle (0${}^{\circ }$) of the left-top quarter-circular region, the intensity of the region is high with parallel polarization filtering. The polarization filter angle of the right-top quarter-circular region is rotated by 90${}^{\circ }$ from the left-top region, so the intensity of the region is dark with cross-polarization filtering. The intensity of the bottom quarter-circular regions is between that of the left-top and right-top regions, and the intensity difference between the two bottom regions is less than the upper regions due to partial parallel (or cross-) polarization filtering with a polarization filter angle of 45${}^{\circ }$ and 135${}^{\circ }$. (b–e) show images for the four polarization states, which were generated by separately sampling pixels in the four quarter-circular regions of each microlens image shown in the raw FPLF photograph in (a).

It is known that measurement of three or more different polarization states allows us to construct a Stokes vector, which characterizes the polarization state of incident light [17]. By employing the method of Riviere et al., we can obtain intensity under cross- and parallel polarization states from the measured Stokes vector. Their method estimates the intensity of cross- and parallel polarization states by solving Mueller calculus [34] associated with the Stokes vector under partially polarized illumination imaged at the Brewster angle of incidence. Since direct sunlight in open sky is partially linearly polarized, we separated diffuse and specular reflectance for the Persian relief by employing the same approach of the near Brewster angle measurement and single-shot light-field sampling, as shown in Figure 5f,g. Note that Riviere et al. [17] measured three polarization states at 0, 45 and 90${}^{\circ }$ to solve an equation with three unknowns, which are then employed for computing diffuse and specular reflectance. We found that solving the equation with four measurements (acquired in a single-shot) with an overdetermined condition yields better SNR and uniformity in separated diffuse and specular reflectance, as shown in Figure 5h,i.

## 5. Layered Reflectance Separation

**Figure 6:**
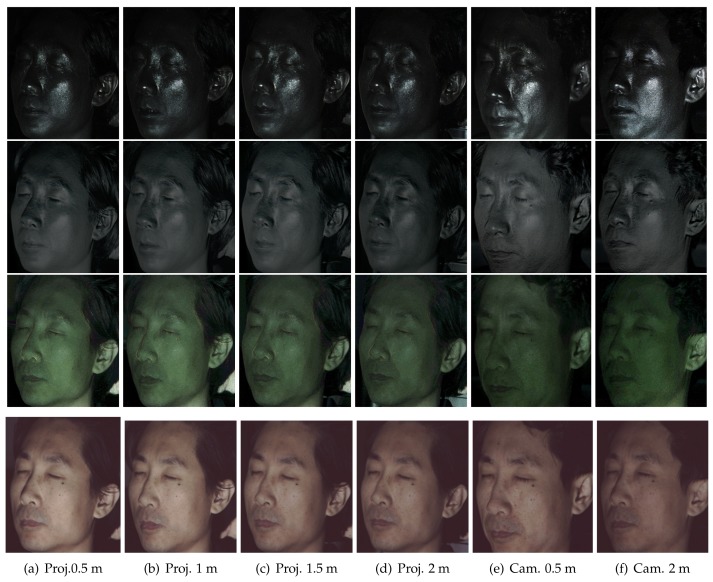
Angular sampling process of the TPLF and FPLF cameras for layered reflectance. (**a**,**c**) Ray diagram through a two-way (**a**) and a four-way polarizer (**c**). (**b**,**d**) Modelling of pixel allocation in a microlens image for a TPLF (**b**) ((**c**) Eurographics Association 2016) and a FPLF camera (**d** [9]).

**Figure 7:**
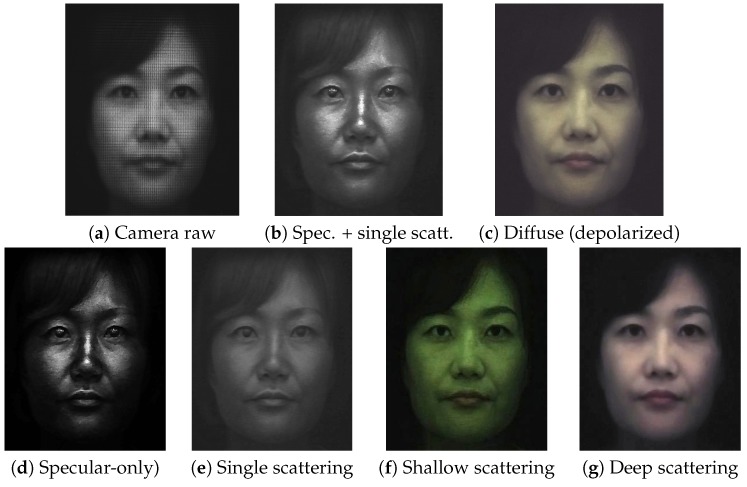
Single-shot layered reflectance separation using the proposed TPLF camera. (**a**) A raw photograph taken by the TPLF camera under uniform (hemi-spherical) illumination (circular microlens pixels visible in the zoomed-in view). (**b**,**c**) Standard polarization preserving (specular + single scatter) and depolarized (diffuse) reflectance components separated with light-field sampling. (**d**,**e**) Specular-only and single scattering further separated from the polarization preserving component (**b**). (**f**,**g**) Shallow and deep scattering components further separated from diffuse component (**c**). Note that (**d**–**g**) present results of layered reflectance separation comparable to the multi-shot technique of Ghosh et al. [8] ((**c**) Eurographics Association 2016 [9]).

The previous sections described diffuse-specular separation using respective sampling of cross- and parallel-polarized semi-circular regions of a microlens in our TPLF and FPLF cameras. In this section, we now describe a novel computational method to obtain layered reflectance separation from a single-shot photograph using angular light-field sampling. It should be noted that in skin, the specular component separated with polarization difference imaging is actually also mixed with some single scattering, which also preserves polarization [8]. This corresponds to the “specular” values of each microlens image in our TPLF camera photograph, as shown in Figure 6b, which are obtained after difference imaging of the lower and upper semi-circular regions of the microlens and include contributions of specular reflectance and single scattering. Similarly, the diffuse values in the upper semi-circular region of the microlens include contributions of both shallow and deep subsurface scattering. We make the observation that our TPLF camera allows us to angularly sample these various reflectance functions in a single photograph. As depicted in Figure 6a, these reflectance functions have increasingly wider reflection lobes ordered as follows: specular reflectance has a sharper lobe than single scattering in the polarization preserving component, and both shallow and deep scattering have wider reflectance lobes, as they are the result of multiple subsurface scattering. Among these, deep scattering has a wider lobe than shallow scattering due to a greater number of subsurface scattering events. Now, the circularly arranged pixels of the microlens angularly sample these reflectance functions as follows: the brighter pixels within each semi-circular region sample both narrow and wide reflectance lobes, while the darker pixels sample only the wider reflectance lobes, which have lower peaks. This motivates us to propose an angular sampling method to separate these various layered reflectance components. We first rely on the observation that specular rays are much brighter than single scattered rays in skin. Hence, we propose separation of these two components by separately sampling high and low intensity values in the polarization preserving component. Each semi-circular region inside a microlens of the Lytro Red Hot camera observes a total of 9×4 = 36 values. We sort these observed values according to their brightness and employ a threshold for the separation. We empirically found averaging the brightest 30% values as specular reflection and averaging the remaining (darker) 70% values as the single scattering component gave good results in practice (see Figure 7d,e).

Next, we assume that deep scattering has a wider angular lobe than shallow scattering in the diffuse component since it scatters further out spatially. Hence, we model deep and shallow scattering components in the diffuse-only region of a microlens image as shown in Figure 6b: darker (outer) pixels correspond to only deep scattering, while brighter (inner) pixels contain both shallow and deep scattering. This modelling assumes that the lobe of shallow scattering is narrow enough not to contribute to the entire diffuse-only region of the microlens. We sample pixels in the darker outer region of each microlens image and average them for generating a deep scattering image, as shown in Figure 7g. Subtracting the deep scattering value from shallow + deep scattering pixels in the brighter inner region generated the shallow scattering image in Figure 7f. We empirically found that using the sampling ratio of the darkest 40% values for estimating deep scattering and the brightest 60% values for estimating shallow + deep provided qualitatively similar results of separation compared to those reported by Ghosh et al. [8], with similar colour tones and scattering amount in each component. Finally, in order to employ the separated shallow and deep scattering components as albedos for layered rendering, we need one additional step of radiometric calibration to ensure that the sum of the two separated shallow and deep albedos matches the total diffuse reflectance albedo in order to ensure that the separation is additive, similar to the separation of [8].

Note that our assumptions about the width of the reflectance lobes is supported by the modelling of Ghosh et al. [8], where they applied the multipole diffusion model [35] for shallow scattering and the dipole diffusion model [36] for deep scattering to model epidermal and dermal scattering approximately. They measured scattering profiles with projected circular dot patterns and fitted the measurements to these diffusion models. While the outer two thirds region of each projected pattern was fitted accurately with the deep scattering model, the inner one-third region was not because of the additional shallow scattering component. Differences in lobe widths of shallow and deep scattering are also supported by the Henyey–Greenstein phase function [37] where the forward scattering parameter g is (0.90, 0.87, 0.85) and (0.85, 0.81, 0.77) for epidermal and dermal layers, respectively, in (R, G, B) [38]. Since the smaller parameter values make the lobe wider, deep scattering occurring at a dermal layer is assumed to have a wider lobe. Therefore, considering the modelled layer thicknesses for epidermal (0.001–0.25 mm) and dermal layers (1–4 mm) in the literature, the radiative transport equation implies that the thicker layer makes the scattering lobe wider [39].

We can extend the layered reflectance separation of TPLF imaging for a FPLF camera shown in Figure 2b under polarized illumination at an arbitrary angle. Figure 6c,d shows how layered reflectance is separately acquired through four polarization filters in a FPLF camera. The diagram (c) assumes the polarization angle of reflected light as 0${}^{\circ }$, so the left-top quarter-circular region of a microlens image, (d), is acquired through parallel polarization with a 0${}^{\circ }$ polarization filter. The left-top quarter-circular region is just the same as the bottom semicircular region of the TPLF imaging shown in Figure 6b, providing diffuse and specular components. The right-top quarter-circular region in (d) undergoes cross-polarization the same as the upper semicircular region of the TPLF imaging, providing the diffuse-only component. The bottom quarter-circular regions in (d) provide diffuse and partial specular components with partial cross-polarization filtering.

**Figure 8:**
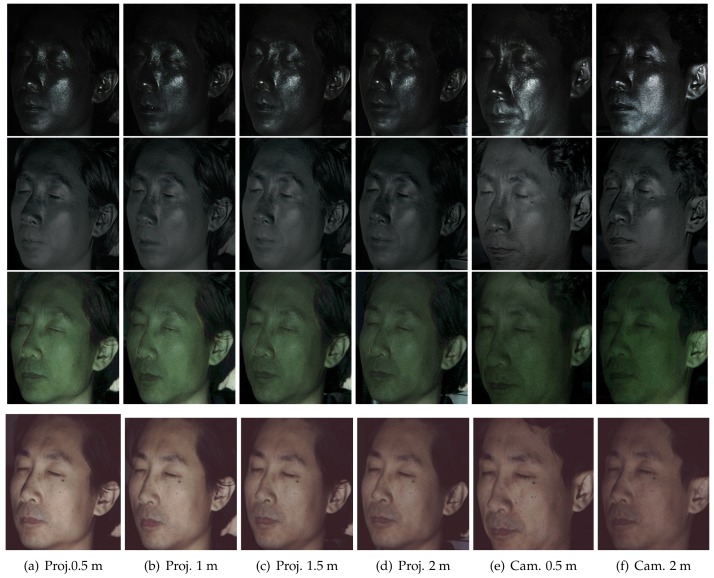
Layered reflectance separation (specular-only, single, shallow and deep scattering from top to bottom) depending on various imaging conditions. A Lytro Illum camera for TPLF imaging was placed at a 1.5-m distance from the subject, and an LCD projector attached with a linear polarization filter was placed at 0.5, 1, 1.5 and 2 m from first to fourth column. The LCD projector was placed at a 1-m distance from the subject, and the Lytro Illum camera was placed at 0.5 m (fifth column) and 2 m (sixth column).

The same intensity-based sampling method of a TPLF camera is applied to each quarter circular region of a microlens image in a FPLF photograph to separately generate layered reflectance images under polarized illumination under an arbitrary angle. For specular-albedo, we generate four light-field images by sampling the brightest 30% pixels in each quarter circular region of a microlens image in a FPLF photograph. By applying the method of Riviere et al. [17], we estimate the intensity of parallel and cross-polarization with the four light-field images. Subtraction of the two intensity values per each pixel gives a specular-only image, as shown in top row of Figure 8. The same procedure with different sampling thresholds applies to generate other layered reflectance albedos. The only difference is that shallow and deep scattering albedos are obtained from the cross-polarized microlens pixels. The same intensity-based threshold ratio is applied to each RGB channel of a TPLF or FPLF photograph. Thus, spectral characteristics of an object such as colour and reflectivity do not affect the proposed separation method. as shown in the eyes of the mannequin in Figure 3 and the eyes of the subject in Figure 7f,g with extreme black and white colours. The dynamic range of the sensor can in principle affect the quality of the separation. This is however somewhat mitigated by our employment of the intensity threshold ratio, which allows sampling of pixels in the relative intensity range. Thus, each layered reflectance albedo has the same relative dynamic range as the raw sensor modulo any quantization errors.

**Figure 9:**
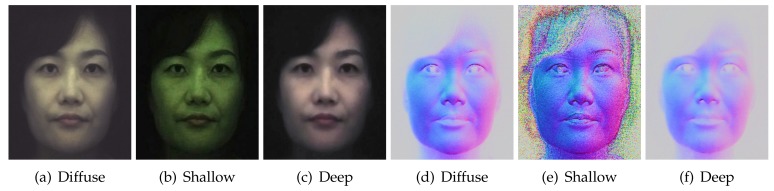
Comparison of albedos (**a**–**c**) and normal maps (**d**–**f**) for the diffuse component and separated shallow and deep scattering ((**c**) Eurographics Association 2016 [9]).

**Figure 10:**
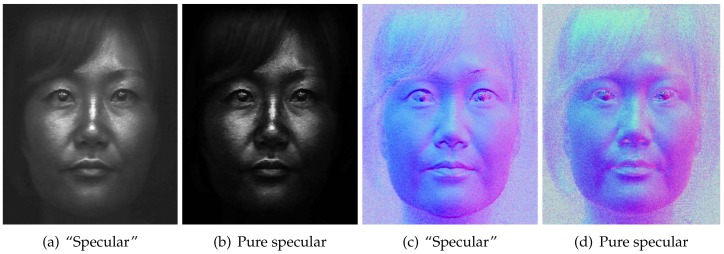
Removal of single scattering from specular reflectance in the polarization preserving component (**a**) for novel computation of pure specular albedo and photometric normals (**b**). Top row: specular albedo. Bottom row: specular normals ((**c**) Eurographics Association 2016 [9]).

Besides separating layered reflectance albedos under uniform polarized illumination, we go further than previous work to separate diffuse normals additionally into shallow and deep scattering normal maps (Figure 9) by applying our novel angular domain light-field sampling to polarized spherical gradient illumination. As can be seen, the shallow scattering normals contain more surface detail than the regular diffuse normals estimated with spherical gradients, while the deep scattering normals are softer and more blurred than the corresponding diffuse normals. We believe this novel layered separation of photometric normals in addition to albedo separation can be very useful for real-time rendering of layered skin reflectance with a hybrid normal rendering approach. Figure 10 demonstrates another similar separation where we remove single scattering from the polarization-preserving component to estimate pure specular albedo and, for the first time, a pure specular normal map (b). Note that specular normal maps have been used to estimate accurate high frequency skin mesostructure detail for high-end facial capture [27,29]. However, “specular” normals (a) as acquired by Ma and Ghosh et al. have some single scattering mixed with the signal, which acts as a small blur kernel on the specular surface detail and can also slightly bend the true orientation of the specular normal. Removal of this single scattering from the data used to compute pure specular normals has the potential for further increasing the accuracy and resolution of facial geometry reconstruction.

## 6. Results

This section describes validation of our method in various imaging conditions. Figure 8 presents the results of layered reflectance separation when the position of a TPLF camera or a light source was changed. We used an LCD projector, an EUG WXGA LCD projector in 5000 Lumens, attached with a linear polarization filter for polarized illumination and a Lytro Illum camera for TPLF imaging. Figure 8a–d shows the results depending on the distance between a subject and a light source when the TPLF camera was placed at a fixed position, 1.5 m from the subject. The distance between a subject and a light source is directly related to the intensity of reflected light. The shallow and deep scattering images from left to right clearly show that the intensity of scattering drops as the distance between a subject and a light source increases from 0.5 m–2 m. It is interesting to see that the slight change of illumination energy is more noticeable in scattering reflection than specular reflection. Despite the change of reflection intensity, we found that the quality of layered reflectance separation is not significantly affected in specular-only, single scattering, shallow scattering and deep scattering, as shown in Figure 8. Thus, this experiment validates that the distance to the light source has a minor influence on the performance of layered reflectance separation with TPLF imaging.

**Figure 11:**
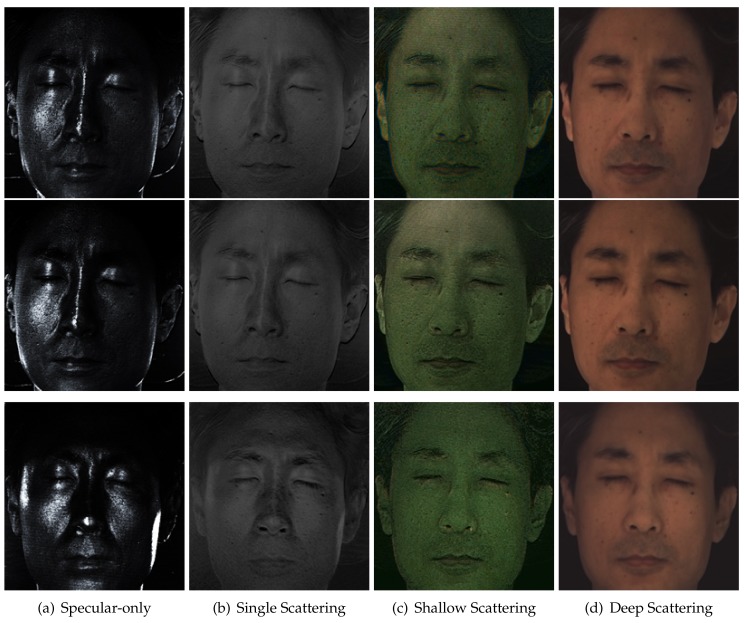
Layered reflectance separation depending on distance between a subject and a Lytro Illum camera for TPLF imaging under polarized spherical illumination. The subject was placed inside a spherical light stage at a near (top row), a middle (middle row) and a far distance (bottom row).

Figure 8e,f presents the results of layered reflectance separation depending on the distance between a subject and a TPLF camera. The change of imaging distance can affect both receiving energy of reflected light and the incident angle of reflected rays to the camera lens. Shallow and deep scattering images in Figure 8e,f show intensity drop as imaging distance increases from 0.5 m–2 m. Furthermore, specular and single scattering images show a change of position of strong reflection. However, the results validate that the quality of layered reflectance separation is not affected by the imaging distance. Note that rays reflected from an object point are sensed by a single microlens image consisting of more than 100 pixels with the Lytro Illum camera. This experiment reveals that such a number of pixels provide sufficient information to resolve four kinds of layered reflectance occurring at an object point regardless of imaging distance. Figure 11 presents the results of layered reflectance separation under polarized spherical illumination using a light stage. The subject was placed at a near (0.5 m, top row), a middle (1.0 m, middle row) and a far distance (2 m, bottom row) inside of the light stage from a Lytro Illum camera for TPLF imaging. Similar to the previous experiment on changing camera distance using an LCD projector, we found that camera distance has a minor influence on performance of layered reflectance separation under polarized spherical illumination. Compared to the previous results with an LCD projector, the results with a light stage show more uniform intensity distribution on the entire face in shallow and deep scattering. It is noticeable that prints of nose pads of glasses are visible between the eyes and the nose in only shallow and deep scattering images.

## 7. Applications and Analysis

**Figure 12:**
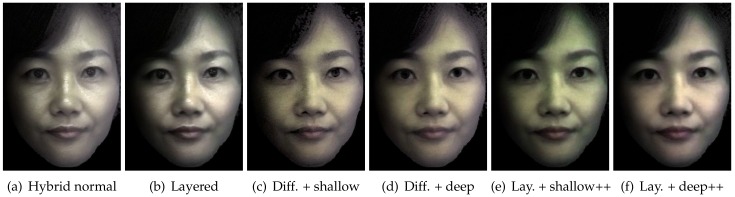
Hybrid normal renderings and editing applications of layered reflectance and normals with data acquired using four photographs under polarized spherical gradient illumination. (**a**) Rendering with separate diffuse and specular normals as proposed by Ma et al. [27]. (**b**) Layered hybrid normal rendering with diffuse reflectance further separated into shallow and deep albedo and normals. (**c**,**d**) Diffuse rendering using shallow scattering normal (**c**) and deep scattering normal (**d**), respectively, for shading the diffuse albedo. (**e**,**f**) Layered hybrid normal rendering with relative enhancement of the shallow scattering albedo (**e**) and relative enhancement of the deep scattering albedo (**f**), respectively.

We now present rendering applications of standard diffuse-specular and layered reflectance separation using our TPLF camera. Figure 12a presents hybrid normal rendering for a female subject (lit by a point light source) according to the method proposed by Ma et al. [27]. Here, the diffuse and specular albedo and normal maps were acquired using four photographs with the TPLF camera under polarized spherical gradient illumination using the LED hemisphere. As can be seen, the specular reflectance highlights the skin surface mesostructure, while the diffuse reflectance has a soft translucent appearance due to blurring of the diffuse normals due to subsurface scattering. Note that the facial geometry for the hybrid normal rendering was acquired separately using multiview stereo with additional DSLR cameras (see Figure 4a). Figure 12b presents a novel layered hybrid normal rendering where the diffuse albedo and normals have been further separated into shallow and deep scattering albedo and normals, respectively. Note that due to the additive property of the separation, the result of layered rendering (b) is very similar to the standard hybrid normal rendering (a) in this case. Figure 12c–f presents a few editing applications of the proposed layered hybrid normal rendering with separated shallow and deep scattering albedo and normals. Here, we have removed the specular layer from the rendering to highlight the edits to the diffuse layer. The top row presents the results of diffuse rendering with the regular diffuse albedo shaded with the shallow scattering normal in (c) and deep scattering normal in (d), respectively. As can be seen, the skin appearance is dry and rougher when shaded with the shallow scattering normal and softer and more translucent when shaded with the deep scattering normal compared to the regular hybrid normal rendering (a). The bottom row presents the results of layered hybrid normal rendering where we have edited the shallow and deep scattering albedos to enhance one component relative to the other. As can be seen, enhancing the shallow scattering albedo (w.r.t. deep) (e) makes the skin appearance more pale and dry, while enhancing the deep scattering albedo (w.r.t. shallow) (f) makes the skin more pink in tone and softer in appearance. The availability of layered reflectance albedos and normals with our TPLF acquisition approach makes such appearance editing operations easily possible.

**Figure 13:**
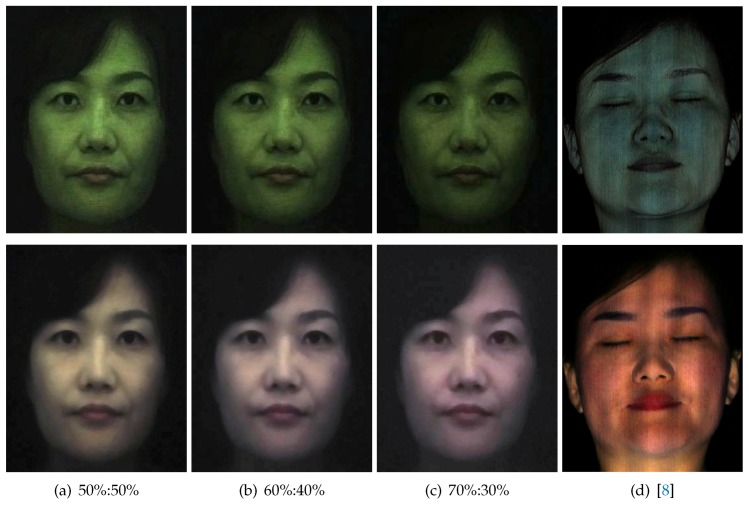
Comparison of shallow and deep separation with single-shot TPLF imaging and the multi-shot technique of Ghosh et al. [8]. (**a**–**c**) Different split ratios of intensity averaging (brighter (shallow + deep)%:darker (deep)%) with TPLF imaging. (**d**) Separation with phase-shifted structured lighting patterns.

Figure 13 presents the results of shallow and deep scattering separation obtained with various intensity thresholds for light-field sampling and compares these single-shot separation results with that obtained using the computational illumination technique of Ghosh et al. [8] (Figure 13d), which uses cross-polarized phase-shifted high frequency structured lighting patterns. As can be seen, our shallow and deep scattering albedo separation with single-shot TPLF imaging achieves similar qualitative results of layered separation compared to the multi-shot technique of [8]. Here, we found the intensity threshold of 60%:40% (brighter 60% values used to estimate shallow + deep, darker 40% used to estimate deep) for the separation of shallow and deep scattering to have the most qualitative similarity to structured lighting-based separation. Note that the multi-shot result was somewhat degraded in comparison to the original paper. This is due to subtle subject motion artefacts during the four shots required to acquire the response to phase-shifted stripes. We employed a commodity Canon EOS 650D camera with sequential triggering to acquire the four shots, while Ghosh et al. [8] used a special head stabilization rig and fast capture with burst mode photography (using a high-end Canon 1D Mark III camera) to prevent such motion artefacts. This further highlights the advantage of our proposed single-shot method.

One advantage of our method is that we employ uniform spherical illumination for separation, which is more suitable for albedo estimation than frontal projector illumination required for structured lighting. The projector illumination also impacts subject comfort during acquisition: the subject had to keep very still and their eyes closed during acquisition of the structured lighting patterns for the separation. We, however, note that there are some visible colour differences between the results of separation with the TPLF camera and separation with structured lighting. These colour differences can be mainly attributed to differences in colour temperatures of the two respective illumination systems (LED hemisphere vs. projector), as well as differences in respective camera colour response curves (the data for structured light-based separation was acquired using a Canon DSRL camera). The colour reproduction with the Lytro camera is not as vivid, because it is based on our own implementation of demosaicing, which may not be optimal.

**Figure 14:**
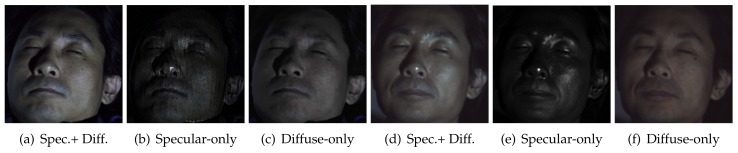
Comparison of specular and diffuse separation with the multi-shot technique of Nayar et al. [11] (**a**–**c**) and single-shot TPLF imaging (**d**–**f**). The same Lytro Illum camera and light source, the EUG WXGA LCD projector, were used for imaging.

Figure 14 compares specular and diffuse separation with single-shot TPLF imaging and the multi-shot direct-indirect separation technique of Nayar et al. [11]. For a fair comparison, we used the same Lytro Illum camera and light source, an EUG WXGA LCD projector for both cases. We took four shots with a normal Lytro Illum camera while illuminating the subject’s face with shifted high-frequency stripe patterns for the multi-shot technique. Then, for single-shot TPLF imaging, we inserted two linear polarizers in vertical and horizontal directions into the Lytro Illum camera at the same position shown in Figure 2b. The results also support how the results of the multi-shot technique are degraded by motion and projection artefacts, especially in the specular-only image; see Figure 14b. Furthermore, the diffuse-only image of the multi-shot technique (Figure 14c) shows inaccurate separation with visible specular components near the left eye.

**Figure 15:**
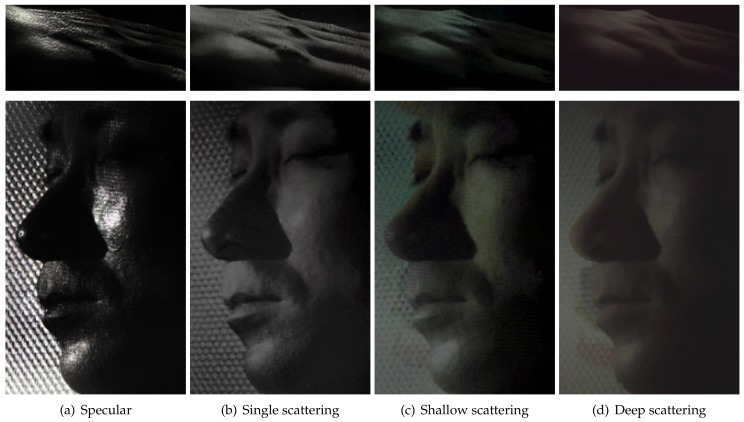
Layered reflectance separation over a hand and a face with single-shot FPLF imaging under sunlight.

We further demonstrate separation of layered reflectance from human skin as described in Section 5 under uncontrolled sunlight with partially polarized illumination at an arbitrary angle using the proposed FPLF imaging. Figure 15 shows layered reflectance of a hand and a face under sunlight incident on the surface near Brewster angle of incidence, which was acquired from a single-shot FPLF photograph. Since sunlight is only partially polarized, note that some portion of specular components was not completed removed in shallow and deep scattering images, especially the cheek and chin regions of the face, due to the high curvature of these regions.

**Figure 16:**
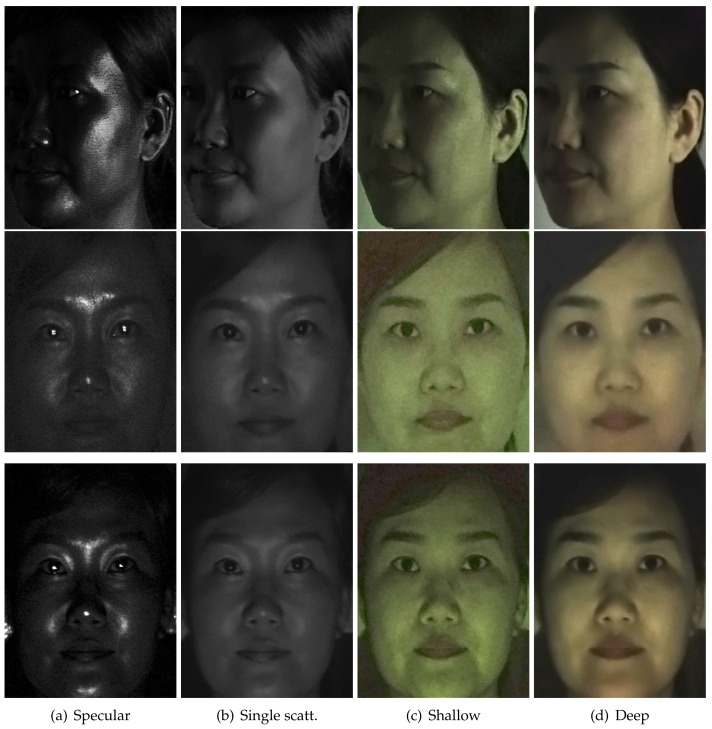
Layered reflectance separation with single-shot TPLF imaging under various illumination conditions. Top row: uniform LCD panel illumination. Centre row: four-point lights. Bottom row: single-point light.

Finally, we analyse the quality of layered reflectance separation with TPLF imaging under various other illumination setups that are commonly available. Figure 16 presents the results of single-shot layered reflectance separation on a subject’s face lit from the side with uniform illumination from a 32${}^{\prime \prime }$ desktop LCD panel (top row), a set of four point light sources (with two on each side) approximating a typical photometric stereo setup (centre row) and finally lit with only a single point light source from the front (bottom row). Here, we simply used smartphone LED flashes as the point light sources in our experiment. Note that the LCD panel we employed already emits (vertical) linearly polarized illumination, so we did not need to explicitly polarize it for the measurement. We mounted plastic linear polarizer sheets (vertically oriented) in front of the phone LED flashes for the other measurements. Among these, employing uniform illumination emitted by the LCD panel resulted in the highest quality of separation, particularly for shallow and deep scattering. Such an illumination setup could also be used in conjunction with passive facial capture systems such as that employed by Bradley et al. [31]. Furthermore, our method also achieves good qualitative layered reflectance separation under just a set of point light sources (and even with a single point light), which has not been previously demonstrated. We believe this can be very useful for appearance capture with commodity facial capture setups. We however note that the separation results with the point light sources suffer from a poorer signal-to-noise ratio due to the larger dynamic range between the specular highlight and the diffuse reflectance compared to when using spherical or extended (LCD) illumination.

### Limitations and Discussion

Since our TPLF and FPLF photography is based on light-field imaging, it suffers from the classical issue of resolution trade-off with a light-field camera. However, the good news is that light-field camera resolution is rapidly increasing, e.g., the Lytro Illum used for our experiment now has a 2K spatial resolution. The Lytro camera does not provide an official way to manipulate the raw light-field photograph, so we implemented our own microlens sampling and colour demosaicing pipeline. This clearly leads to sub-optimal results, as we could not obtain precise information on microlens position for precise pixel sampling, and also, colours were not reproduced as vividly as desirable. The colour reproduction can be improved by measuring a colour chart with the TPLF camera and transforming measured colour values to the sRGB colour space. Our separation with TPLF imaging assumes that the incident illumination is polarized with its axis either vertically or horizontally oriented in order to match the orientation of our two-way polarizer. Fortunately, this is not very hard to achieve in practice with many illumination setups. Our FPLF imaging is not bounded by this assumption since it allows us to estimate parallel and cross-polarization states under polarized illumination at an arbitrary angle. The advantage of FPLF imaging compared to TPLF imaging is enhanced flexibility with respect to the incident illumination. While TPLF imaging requires aligned orientation of polarized illumination, FPLF imaging works with uncontrolled and arbitrary partially-polarized illumination, such as sunlight. Thus, only FPLF imaging can be applied for outdoor applications. However, separated reflectance images obtained using TPLF imaging have higher SNR than those obtained with FPLF imaging since TPLF imaging samples twice the number of pixels in the microlens compared to FPLF imaging. Thus, there exists a trade-off between flexibility and SNR in the number of filters employed for polarized light-field imaging. The ideal number of polarization filters is the minimum number of filters required for a particular application. The implementation of a TPLF camera using a Lytro Red Hot camera has a restricted dynamic range compared to DSLR cameras, which is why it is currently more suitable for acquisition with spherical or extended illumination compared to point light sources. However, we expect this dynamic range issue to be resolved with improvements in light-field camera technology in association with HDR imaging. There is also the possibility of misalignment error with manual attachment of a two-way or four-way polarizer into a light-field camera. This could be resolved in future work by employing an imaging sensor with an inbuilt linear polarizer (e.g., Sony’s IMX250MZR).

We currently empirically set the intensity thresholds for layered reflectance separation. Further research would be required to relate such separation thresholds to physiological characteristics of reflection and scattering of light in various layers of skin. We also simply render skin appearance with a hybrid normal rendering approach using the separated reflectance albedos and photometric normals. However, many rendering systems instead implement full blown subsurface scattering simulations to achieve translucency effects. Besides scattering albedos, such a rendering approach requires an estimate of translucency (diffuse mean free path), which we do not currently measure. However, the approach of Zhu et al. [40] could be employed with our data to estimate per pixel translucency from four measurements under polarized spherical gradient illumination. Furthermore, our separation of diffuse albedo and normals into shallow and deep scattering albedo and normals might enable estimation of layered translucency parameters for rendering shallow and deep scattering using such an approach. Our imaging approach for layered reflectance separation may also have applications in other domains such as cosmetics where the technique could be applied to separate a cosmetic layer from the underlying skin reflectance or applications in industrial or cultural heritage sectors for separation of layered materials such as paints or pigments.

## 8. Conclusions

To summarize, we propose a novel computational photography method for single-shot separation of diffuse and specular reflectance, as well as more fine-grained separation of layered reflectance using a two-way polarized and a four-way polarized light-field camera. Our imaging approach allows estimation of diffuse, specular, single scattering, shallow scattering and deep scattering albedos from a single photograph captured under various forms of controlled polarized and partially-polarized illumination. We further demonstrate novel layered separation of photometric normals acquired using polarized spherical gradient illumination with just three additional photographs. Thus, our approach significantly reduces the number of required measurements for layered reflectance acquisition compared to existing multi-shot techniques, while enabling novel appearance editing applications with layered reflectance and normal maps. We also demonstrate our single-shot reflectance separation technique to work well in practice with a variety of commodity illumination setups and even under sunlight. This can be very useful for appearance acquisition with commodity facial capture setups that employ passive imaging. Our method could be extended in the future to estimate more complete reflectance information such as parameters to describe a full BRDF or a BSSRDF with a combination of appropriate light-field filtering and controlled illumination. The proposed layered reflectance separation approach could have applications in various other domains such as dermatology/cosmetics, automotive or cultural heritage applications.

## Abbreviations

The following abbreviations are used in this manuscript:

TPLF	two-way polarized light-field
FPLF	four-way polarized light-field
